# Post-Traumatic Stress Disorder among civilians in Indian-administered Kashmir: a systematic review

**DOI:** 10.3389/fpsyt.2026.1919292

**Published:** 2026-09-15

**Authors:** Tamkeen Sakeena, Aneeza Pervez, Cecilie Lolansen

**Affiliations:** 1 School of Psychology, University of Nottingham, Nottingham, United Kingdom; 2 Clare Hall Fellow, Faculty of Education, University of Cambridge, Cambridge, United Kingdom

**Keywords:** Kashmir, PTSD, trauma, civilian trauma, armed conflict, militarisation

## Abstract

**Purpose:**

Prolonged exposure to armed conflict is associated with elevated rates of PTSD in civilian populations, such as Indian-administered Kashmir. This review aimed to systematically synthesise research on prevalence, risk factors, and correlates of PTSD among civilians in this setting and their association with armed conflict exposure.

**Methods:**

A systematic review following PRISMA guidelines was conducted for empirical studies published up to 2025. Sixteen quantitative studies met the inclusion criteria. Due to heterogeneity in study design, sampling, and PTSD assessment, findings were synthesised narratively.

**Results:**

Reported PTSD prevalence varied across studies. Among community-based adult samples, prevalence ranged from 15.9% to 24.5%, with higher screening-based estimates in selected populations, including college students (up to 49.8%) and children (up to 40%) meeting a PCL-C threshold for probable PTSD. Higher prevalence was observed among women, socioeconomically disadvantaged groups, orphans, and individuals with direct conflict-related injuries. Several studies reported dose-response relationships between cumulative trauma exposure and PTSD, with PTSD symptoms commonly reported even years after exposure and comorbid with depression, anxiety, and substance misuse. Several limitations of existing literature were identified, such as considerable variation in study quality and reliance on cross-sectional research.

**Conclusion:**

This review reveals substantial levels of PTSD among civilians in Indian-administered Kashmir. It highlights variability driven by methodological differences in measurement, sampling, and exposure assessment. Greater consistency in assessment and study design and more longitudinal studies are needed to strengthen psychiatric epidemiological evidence in armed conflict settings to inform psychiatric support approaches in prolonged armed conflict settings. The findings highlight how the scale of need for psychiatric care in Kashmir exceeds capacity, prompting a need to explore possible low-resource interventions options such as community-based and task-shared approaches, options which may also be applicable in other militarised, low-resource settings.

**Systematic Review Registration:**

https://osf.io/3ztxq.

## Introduction

Across the lifespan, individuals are exposed to stressors and challenging events. However, some have the potential to develop into clinically significant distress, such as Post-traumatic Stress Disorder (PTSD). PTSD is a mental health disorder that emerges after exposure to traumatic events involving experiences such as real or threatened death, serious injury or sexual violence (1) and is characterised by symptoms, such as heightened arousal, intrusive memories, avoidance behaviours, and negative alterations in mood and cognition (2, 3). While PTSD can occur following a wide range of events and exposures, it is particularly prevalent in populations exposed to armed conflict, where people are exposed to multiple traumatic experiences, often chronically (4).

People living in conflict-ridden areas face an increased risk of developing PTSD and other mental health disorders (4). When civilians are exposed to traumatising events like torture, sexual violence, forced displacement, and are made witness to the destruction of their homes and properties, it significantly contributes to their psychological vulnerability and is associated with considerable psychiatric morbidities (3, 5). A recent systematic review and meta-analysis found that approximately 24% of civilians in conflict-ridden areas meet the diagnostic criteria for PTSD, and 26% are found with symptoms of depression (4). Furthermore, among migrants who are exposed to armed conflict, PTSD has been reported to be as high as 31% (6). While the estimated PTSD prevalence in the general population varies greatly depending on methodology and population (7–9), the rates in areas of conflict and in populations exposed to armed conflict are elevated. In addition to PTSD, a third of individuals in conflict zones face other psychiatric challenges, including depression (10–12), anxiety (11, 12), and suicidal ideation (13, 14).

When exploring prevalence, it is important to consider factors which affect prevalence. Some general population research suggests that women have greater risk of developing PTSD (8, 15, 16). Evidence from Afghanistan reveals that war-related violence, such as injury, bereavement and displacement, is strongly associated with elevated rates of PTSD among women (17). Research has also implicated other factors in PTSD risk, such as socioeconomic status (10, 18, 19), personal education (10, 18), parental education (19), and cumulative trauma exposure (10). While accounting for such a range of factors can be challenging, it is important to consider the complexities of risk factors and experiences to provide a nuanced exploration of PTSD and its symptomatology.

### The context of Kashmir

The Kashmir valley, a Muslim-majority region within Indian occupied Kashmir, provides a distinctive setting for examining the relationship between prolonged armed conflict and PTSD. The conflict in Kashmir is often referred to as “South Asia’s longest-running conflict” (20) and “the world’s most densely militarised place” (21), hosting over 700,000 Indian armed personnel (22, 23). Since the partition of British India in 1947, Kashmir has remained an ‘unfinished business’ between India and Pakistan. This has led to a long and violent geopolitical conflict evinced through territorial disputes and military confrontations (24). In 1989, an armed insurgency erupted in Kashmir against Indian rule and transformed the conflict into a low-intensity war between the local rebels and Indian armed forces. This period was marked by human right violations, extrajudicial killings, forced disappearance, and sexual violence, committed under the guise of emergency legislation (22, 25). The collective effect of decades of militarised governance has resulted in an estimated killings of over 10,000 civilians by the security forces in extrajudicial actions and around 8,000 individuals have disappeared since the beginning of insurgency in 1989 (26).

Kashmir shares prolonged insecurity and civilian exposure to violence with conflict-affected settings such as Afghanistan, Palestine and Syria, so its relevance does not rest on treating these conditions as exclusive to the region. Its distinctiveness lies in the particular convergence of a decades-long territorial conflict, sustained militarisation within everyday civilian spaces, repeated exposure to crossfire, raids, detention and other forms of violence, and the absence of a clearly demarcated post-conflict period (13, 21, 22). Civilian survey research has documented frequent exposure to crossfire, round-up raids, detention, torture and other forms of violence, alongside continuing feelings of insecurity associated with psychological distress (13, 14). The concept of continuous traumatic stress is relevant to this setting because danger remains ongoing and realistic, complicating the distinction between responses to past trauma and vigilance towards present threat (27). Ongoing exposure may therefore influence PTSD prevalence by sustaining distress and limiting opportunities for recovery, while symptom presentation may include heightened vigilance and threat anticipation that arise within conditions of continuing danger. Daily social and economic stressors also form part of the environment in which trauma-related distress develops and is maintained, although their contribution cannot be separated fully from direct conflict exposure in the available cross-sectional evidence (28). The studies reviewed do not permit direct comparison of PTSD prevalence or symptom profiles with Afghanistan, Palestine or Syria. Kashmir nevertheless warrants focused examination because it allows PTSD to be considered within a prolonged configuration of everyday militarisation, continuing civilian exposure and limited opportunities for a stable post-conflict recovery period.

While individual studies have established that Kashmiris are exposed to severe trauma (29–31), a systematic review examining the role of armed conflict on PTSD in civilians in the general civilian population in Kashmir has not been published. Previous meta-analyses have established a high prevalence of PTSD in conflict-ridden areas (4, 6) and multiple empirical studies have shown the widespread traumatic experiences of people living in Kashmir (29, 32). Despite that, a comprehensive examination of how conflict-related exposures shape PTSD in this context remains absent, as previous reviews have either focus on India more broadly (33), on specific populations such as children in Kashmir (34), or in the case of Bhat and colleagues’ (35) systematic review, this was broad in scope and due to its publication date, did not include research published since 2017. Moreover, the available evidence has not been synthesised in a way that considers how cumulative exposure, continuing threat and differences in vulnerability relate to PTSD across civilian populations in Indian-administered Kashmir. For this reason, the present study seeks to explore the prevalence, risk factors, and correlates of PTSD among civilians in this setting to provide a comprehensive assessment of the conditions through which PTSD can occur, its course shaped, and including the most recent empirical findings.

### Conceptual framework

For the purposes of synthesis and interpretation, this review draws on an integrated psychosocial account of conflict-related distress, combining Miller and Rasmussen’s (36) model of war exposure and daily stressors with the concept of continuous traumatic stress (27). Miller and Rasmussen (36) propose that the psychological effects of armed conflict arise through both direct exposure to potentially traumatic events and the social and material stressors generated or intensified by conflict, with daily stressors potentially mediating part of the relationship between war exposure and mental health. This distinction is relevant in Kashmir, where Housen, Lenglet, et al. (28) examined financial stress, family stress and poor physical health as possible mediators of the association between cumulative traumatic exposure and symptoms of PTSD, depression and anxiety. The observed mediation effects were small, providing limited support for an account in which daily stressors explain the relationship between conflict exposure and psychological distress, while showing the value of considering direct traumatic exposure alongside the wider conditions of everyday life.

Continuous traumatic stress provides a complementary way of considering the temporal context in which these relationships occur. Eagle and Kaminer (27) developed the construct to describe psychological responses in settings where serious threat remains realistic, current and potentially recurring, complicating assumptions that traumatic danger is located exclusively in the past. Within this review, these perspectives inform the interpretation of cumulative exposure, chronicity, and vulnerability in related, but distinct ways. Cumulative exposure refers to repeated or multiple forms of conflict-related trauma and their association with PTSD outcomes; evidence concerning chronicity is interpreted within a setting in which threat and exposure may continue rather than assuming a stable post-conflict period; and vulnerability factors are considered in relation to the wider social, economic, developmental and exposure conditions that may shape both psychological risk and opportunities for recovery. This framing does not treat these processes as established causal pathways but provides a basis for interpreting the patterns reported across a predominantly cross-sectional and heterogeneous evidence base.

## Methods

This systematic review was conducted in accordance with the Preferred Reporting Items for Systematic Review and Meta-Analyses (PRISMA) guidelines (37) and was managed using the online systematic review software Covidence (38). The study protocol and methods were preregistered on the Open Science Framework (OSF: https://osf.io/3ztxq/). No deviations from the preregistered protocol were made.

Due to considerable heterogeneity between included studies, meta-analysis (quantitative synthesis) was not considered appropriate. Specifically, studies differed in (a) population: from general community-based adults to college students, children, orphans, conflict-related amputees and health care professionals, populations not directly comparable in terms of baseline risk, (b) study design: including population-representative surveys, retrospective recall studies and small clinical or treatment-seeking case series, each with different sources of bias; (c) PTSD assessment method and diagnostic threshold, including continuously scored instruments (e.g., the Harvard Trauma Questionnaire), screening measures with preset cut-points (e.g., the PTSD Checklist-Civilian), structured clinical diagnostic interviews (e.g., DSM-IV/ICD-10 criteria, the Clinician-Administered PTSD Scale) with each yielding incomparable estimates of the same construct (d) sample sizes that varied from 16 to 5,519 participants, an extent of variability that would yield considerable statistical heterogeneity if an unweighted aggregate effect estimate were generated. Finally, not all previous studies adequately reported the variance information necessary for the calculation of a weighted aggregate effect estimate; therefore, to circumvent the potential for the generation of an unstable and unintelligible statistic, this review is structured as a descriptive narrative synthesis according to PRISMA 2020 guidelines on when meta-analysis might not be appropriate (37).

### Search strategy

Four electronic databases were searched: APA PsycINFO, PubMed, ScienceDirect, and Scopus. The literature search was conducted in July 2025, with the final searches completed on 31 July 2025. Initial search strings were developed to assess sensitivity and relevance across databases and were subsequently refined to produce database-specific final search strategies. Unless otherwise specified within individual database interfaces, no restrictions were placed on year of publication.

The final search strings were as follows:

- APA PsycINFO: (Kashmir*) AND (PTSD OR Trauma*)
- PubMed: Kashmir* AND PTSD
- ScienceDirect: (PTSD OR “post-traumatic stress” OR “psychological distress”) AND Kashmir AND (“armed conflict” OR violence OR militarization)
- Scopus: TITLE-ABS-KEY (Kashmir AND PTSD*)

Broader trauma-related terms were included to maximise search sensitivity; however, studies were eligible for inclusion only if post-traumatic stress disorder (PTSD) was explicitly assessed using a validated diagnostic interview or screening measure. Full database-specific search strategies are provided in Appendix A (online resource).

### Eligibility

Studies were eligible for inclusion if they met the following criteria: (a) original empirical research; (b) published between database inception and July 2025; (c) written in English; (d) conducted among civilian populations residing in the Indian-administered part of Kashmir; and (e) assessed PTSD using a validated diagnostic interview or screening instrument in relation to armed conflict exposure.

Studies were excluded if they did not present original empirical data (e.g. reviews, commentaries, opinion pieces), were not conducted among civilian populations in the Indian-administered part of Kashmir or did not include an explicit assessment of PTSD or its association with armed conflict exposure.

### Study selection

All records identified through database searching were imported into Covidence for screening and management (38). A total of 192 records were identified across the four databases. After removal of duplicates (*N* = 30), 162 records remained for screening.

Studies were screened in two stages. In the first stage, titles and abstracts were screened against the eligibility criteria, resulting in the exclusion of 143 records. In the second stage, the full texts of 19 studies were assessed for eligibility. All full texts were successfully retrieved.

Following full-text assessment, three studies were excluded due to wrong setting (*N* = 1) or inappropriate study design (*N* = 2). A total of 16 studies met the eligibility criteria and were included in the final synthesis. The study selection process is illustrated in the PRISMA flow diagram (37) in **Figure 1**.

**Figure 1:**
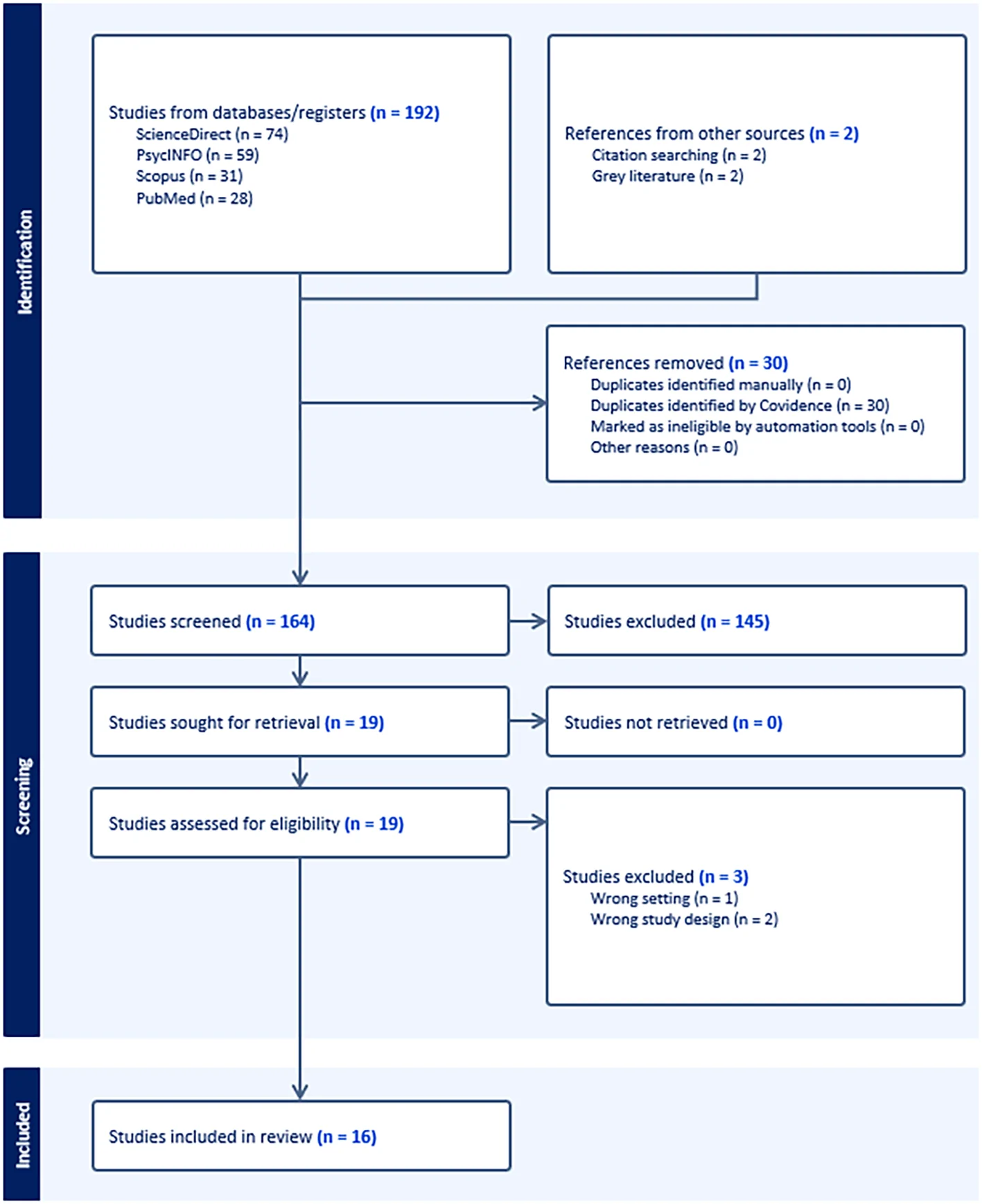
PRISMA-style image showing the study selection process and results for this review.

### Data extraction

Data was extracted using a structured extraction form developed in Covidence in line with the preregistered protocol. Extracted information included author and year of publication, study aim, study location and setting, sample characteristics, exposure to armed conflict, PTSD assessment method, study design, measures used, and primary findings relating to PTSD prevalence and associated factors. Data extraction was conducted by a single reviewer in accordance with the preregistered protocol.

### Quality assessment

The methodological quality of the included studies was assessed using the Standard Quality Assessment Criteria for Evaluating Primary Research Papers from a Variety of Fields developed by Kmet et al. (39). This tool evaluates key methodological domains including study design, sampling strategy, measurement validity, analytic approach, and clarity of reporting. Each study was scored in accordance with the Kmet guidelines, and an overall quality score was calculated by summing item scores and expressing the total as a proportion of the maximum possible score.

Quality assessment was conducted by a single reviewer (TS) in accordance with the preregistered protocol. Quality scores were used to inform interpretation of the findings but were not used as exclusion criteria. Individual study quality scores are reported in Appendix B (online resource).

The quality of methodology of all 16 included studies, appraised based on the criteria established by Kmet et al. (39), varied from 0.45 to 1.00. Of the total number, 10 studies (63%) were found to be of high quality (score ≥ 0.80), comprising principally large, population-representative surveys and psychometrically oriented studies, involving validated methods of testing and proper statistical analysis; two large scale studies by Housen et al. (29, 30) received the highest possible score. Two studies were rated moderate quality (0.60–0.79): Margoob et al. (40), a household community-prevalence survey, and Margoob et al. (41), a study of PTSD following amputation, both single-site studies with more limited statistical adjustment than the higher-scoring studies. Four studies were rated as low quality (< 0.60) with scores ranging from 0.45 to 0.55: a case–control study of children exposed to terrorist attacks (42), and three other clinical samples from 2006 reported by Margoob et al.: treatment-seeking PTSD patients (2006b), conflict-exposed children and adolescents (2006c), and orphaned children (2006d), which were characterised by small, non-representative clinical recruitment and limited reporting of variance or control of confounds. The overall methodological rigour improved over time, with post 2014 published studies more consistently using validated diagnostic instruments, larger or more representative samples and appropriate statistical control. Given this variation, estimates of prevalence from the population-based studies of higher quality should be weighted more than the smaller, lower quality clinical case series when interpreting the data.

### Synthesis of results

Owing to substantial heterogeneity across included studies in terms of design, sampling strategies, populations, PTSD assessment instruments, and outcome reporting, quantitative synthesis through meta-analysis was not appropriate. Accordingly, findings were synthesised using a structured narrative approach, consistent with PRISMA guidance for systematic reviews where statistical pooling is not feasible (37).

Where studies employed reported PTSD prevalence using screening instruments symptom checklist, structured diagnostic interviews or clinician-administered assessments, findings were summarised descriptively and ranges were reported, but results were not statistically combined. Classifications derived from self-report screening thresholds were described as probable PTSD, while the term PTSD diagnosis was reserved for studies in which diagnostic status was established through a structured or clinician-administered assessment. Where measures were analysed continuously, findings were described as PTSD symptom severity. This approach enabled the evidence to be integrated systematically while retaining differences in diagnostic framework, instrument type, outcome definition, sampling context and exposure profile. The narrative synthesis focused on patterns in prevalence, identified vulnerability factors, trauma exposure profiles, and commonly reported correlates without treating methodological distinct estimates as directly equivalent. The conceptual framing outlined earlier, was used to interpret patterns relating to cumulative exposure, chronicity and vulnerability within the narrative synthesis. It did not alter the preregistered search strategy, eligibility criteria or data extraction procedures.

## Results

A total of 16 quantitative studies, published between 2006 and 2025, met the inclusion criteria, and were included in the final synthesis (**Table 1**). The studies varied considerably in design, sample size, population characteristics, and PTSD assessment methods, encompassing community-based surveys, clinical and treatment-seeking samples, and investigations of highly exposed subgroups, including children, amputees, and healthcare professionals. PTSD outcomes were assessed using a range of self-report screening instruments, symptom measures, and structured diagnostic interviews, most commonly the Harvard Trauma Questionnaire, PTSD Checklist-Civilian Version, and DSM-IV-based clinical interviews.

**Table 1:** Characteristics of included studies.

Author (year)	Aim	Location	Sample	Exposure to armed conflict	PTSD assessment	Design	Measures	Primary findings
Basu & Dutta (42)	Examine psychological changes in children exposed to terrorist violence	Srinagar	131 exposed children (1.5-15 yrs) and 90 matched controls	Direct exposure to terrorist bombing	Trauma-related behavioural symptoms assessed via CBCL (non-diagnostic)	Cross-sectional, matched controls	CBCL (parent-report); risk-factor questionnaire	Exposed children showed higher withdrawn, somatic, and anxious/depressed scores; maternal death and cumulative stressors associated with higher symptom levels
Bhat & Rangaiah (32)	Examine the prevalence of post-traumatic stress symptoms and their association with conflict exposure among young adults.	Northern Kashmir	797 conflict-exposed college students (19-24 yrs)	Exposure to conflict-related events	PCL-C, a 17-item DSM-IV-aligned self-report screening measure; probable PTSD defined using a cut-off score of 50	Cross-sectional	Exposure to Kashmir Conflict Checklist; PTSD Checklist–Civilian Version (PCL-C)	49.8% met the study’s PCL-C screening threshold for probable PTSD. Greater cumulative exposure and several specific conflict-related experiences were associated with increased PTSD symptomatology.
Charak et al. (43)	Examine PTSD factor structure and gender differences	Jammu & Kashmir	113 adults displaced by cross-border firing	Displacement and loss due to shelling and firing	DSM-IV-TR PTSD symptom criteria (factor analytic modelling)	Cross-sectional CFA	PTSD Checklist (stressor-specific)	PTSD prevalence ~25%; females scored higher on re-experiencing and anxious arousal
Dar & Iqbal (44)	Examine relationship between PTSD symptoms and post-traumatic growth	Indian-administered Kashmir	120 pellet-gun-injured adults (18-28 yrs)	Direct conflict-related injury	PCL-C/PCL-5	Cross-sectional	PTGI-SF; LEC-5; time-since-trauma	Clinically significant PTSD symptoms common; moderate PTSD associated with higher PTG
Dar et al. (45)	Examine childhood trauma and adult psychiatric outcomes	Kashmir	3,137 adults (572 trauma-exposed)	Childhood conflict-related trauma	MINI (DSM-IV-TR PTSD)	Retrospective population-based	MINI; life-event checklist	PTSD prevalence 24.4% among trauma-exposed; symptoms persisted beyond 10 years
Dar et al. (46)	Examine secondary traumatic stress among healthcare workers	Kashmir	337 healthcare professionals	Secondary exposure through clinical work	STSS	Cross-sectional	STSS; PTGI-SF; ERRI; BTQ	Moderate secondary traumatic stress; intrusive rumination associated with higher STS
Housen et al. (29)	Estimate prevalence of PTSD, anxiety, and depression	Kashmir Valley	5,428 adults	Multiple conflict-related traumatic events	HTQ-16 (probable PTSD)	Population-based survey	HTQ-16; HSCL-25; trauma checklist	Probable PTSD (HTQ cut-off): 19%; dose-response with number of traumatic events
Housen et al. (30)	Examine role of daily stressors in PTSD	Kashmir Valley	5,519 adults	Conflict-related trauma	HTQ-16	Secondary analysis	Daily Stressors Inventory; HSCL-25	Daily stressors partially mediated trauma-PTSD relationship
Margoob et al. (40)	Document trauma exposure and PTSD prevalence	Kashmir Valley	671 adults	Multiple armed conflict events	DSM-IV symptom checklist	Cross-sectional	Trauma checklist; DSM-IV checklist	Lifetime PTSD prevalence 15.9%
Margoob et al. (47)	Describe treatment-seeking PTSD patients	Kashmir	469 diagnosed PTSD patients	High cumulative conflict exposure	HTQ-16; clinical diagnosis	Clinical cross-sectional	HSCL-25; trauma checklist	85.1% met criteria for chronic PTSD (treatment-seeking sample)
Margoob et al. (48)	Examine PTSD symptoms in children and adolescents	Kashmir	56 children (3-16 yrs)	Witnessing and direct conflict exposure	DSM-IV PTSD diagnosis	Cross-sectional	Clinical interview; symptom clusters	High levels of re-experiencing, avoidance, hyperarousal; acute onset common
Margoob et al. (49)	Examine psychiatric disorders among orphans	Srinagar	76 orphaned children	Parental death due to conflict	DSM-IV-TR	Clinical survey	MINI-KID; trauma history	PTSD present in 40.6%; multiple comorbid diagnoses common
Margoob et al. (41)	Estimate PTSD following amputation	Kashmir	16 amputees	Conflict-related injury	CAPS	Cross-sectional	Structured clinical interview	PTSD prevalence 66%; higher among conflict-related amputations
Shoib et al. (50)	Estimate PTSD prevalence and comorbidity	Kashmir	3,400 adults	Family exposure to armed conflict	DSM-IV-TR; CAPS	Cross-sectional	GHQ; CAPS	PTSD prevalence 3.76%; depression/anxiety common
Wani & Margoob (51)	Examine psychiatric morbidity in relatives	Srinagar	221 first-degree relatives	Indirect exposure via PTSD patients	DSM-IV/ICD-10	Cross-sectional	MINI-Plus; CAPS	62% psychiatric morbidity; PTSD most common
Yaswi & Haque (52)	To examine prevalence of PTSD symptoms, depression, and coping among conflict-affected adults.	Kashmir Valley, India	80 adults (mean age 33.1 years); recruited from hospital OPD, university, rural households.	Direct or indirect exposure to armed conflict (crossfire, killings, raids, disappearances).	Through Everstine Trauma Response Index–Adapted (ETRI), self-report scale measuring trauma exposure, PTSD symptoms, and symptom duration.	Cross-sectional community study.	ETRI (35 items); Beck Depression Inventory; Coping Resources Inventory.	Directly exposed group had significantly higher PTSD symptoms (M = 167.42) vs indirect (M = 126.69); also higher depression and lower coping.

Given the heterogeneity across studies, findings were synthesised narratively and organised into four analytically distinct domains: (1) distribution and prevalence of PTSD, (2) exposure pathways and vulnerability factors, (3) comorbidity and broader psychosocial consequences, and (4) temporal course and chronicity of PTSD. Together, these domains capture patterns in the occurrence, determinants, and persistence of PTSD among civilian populations living under prolonged armed conflict in Indian-administered Kashmir.

### Distribution and prevalence of PTSD

Across the included studies, estimates of PTSD among civilian populations in Indian-administered Kashmir varied markedly according to sampling context, exposure profile, and assessment method. In adult community samples, reported prevalence of PTSD or probable PTSD ranged from 3.76% to approximately one-quarter, with the lowest estimate reported by Shoib et al. using DSM-IV-TR criteria confirmed clinically (50). Higher community-level estimates were reported in surveys using validated screening instruments, with probable PTSD identified in 15.9% of adults in a household survey (31) and 19% in a large-scale survey using the Harvard Trauma Questionnaire (29).

Among adults with conflict-related amputations, 66% met criteria for PTSD following structured clinical assessment (41). In paediatric samples, prevalence estimates were not consistently reported; however, orphaned children assessed clinically showed a PTSD prevalence of 40.6% (49), and conflict-exposed children assessed using DSM-IV criteria demonstrated high symptom burden (48). Extremely high prevalence was reported in treatment-seeking populations, with 85.1% of adults presenting for PTSD care meeting criteria for chronic PTSD (47). Given the clinical nature of this sample, this estimate is not considered representative of population-level prevalence.

Among young adults, a cross-sectional study of college students found that 49.8% met the study’s PCL-C threshold for probable PTSD. The proportion meeting this threshold increased with cumulative exposure to conflict-related events (32). In addition, children exposed to terrorist violence demonstrated elevated levels of trauma-related behavioural symptoms, including withdrawal and anxiety, relative to non-exposed controls (42).

### Exposure pathways and vulnerability factors

Across studies, direct exposure to armed conflict was consistently associated with higher levels of PTSD or PTSD symptom burden. Exposure pathways captured in the included literature included conflict-related injury, displacement and loss, and witnessing or experiencing violence (41, 42, 48). In samples characterised by direct injury, PTSD prevalence was high, including among conflict-related amputees (41). Displacement and loss were also linked to elevated PTSD symptomatology in adult conflict survivors, including displaced populations assessed for PTSD symptom structure and gender differences (43).

Beyond single-event trauma, several studies reported evidence consistent with a dose-response pattern, whereby greater cumulative exposure was associated with higher PTSD prevalence or symptom severity. It is possible that repeated exposure to both events and associative cues can results in fear conditioning (53), and that this conditioning combined with chronic exposure to stressors reinforces PTSD symptoms through impairment of fear extinction processes (54), as reflected in our dose-relationship findings. Similarly, the nature of repeated exposure and ongoing militarisation of Indian-administered Kashmir may mean that coping resources become scarce. This is important to consider when assessing vulnerability, as research has showing coping strategies and availability of coping resources can affect symptoms and recovery course (55, 56).

In a large population-based survey using the Harvard Trauma Questionnaire, probable PTSD increased as the number of conflict-related traumatic events increased (29). Similarly, among college students, the proportion meeting the PCL-C threshold for probable PTSD increased with cumulative exposure to conflict-related events, while higher symptom scores were also associated with several forms of exposure (32).

Higher PTSD prevalence or symptom severity was observed among women (29, 43) and socioeconomic disadvantage, including lower socioeconomic position and unemployment, was associated with higher PTSD outcomes in community samples (29, 45); however, one study identified poorer coping in men compared to women (52). In paediatric samples, conflict-related family loss was a key vulnerability marker, with orphaned children showing high psychiatric morbidity, including PTSD (41), and children exposed to terrorist violence exhibiting elevated trauma-related behavioural symptoms compared with non-exposed controls (42).

Evidence also suggested that trauma-related psychological impacts extended beyond directly exposed individuals through secondary exposure pathways. Healthcare professionals reported secondary traumatic stress and trauma-related cognitive responses in relation to occupational exposure (46). In addition, first-degree relatives of PTSD patients showed high levels of psychiatric morbidity, with PTSD the most frequently reported diagnosis (51).

### Comorbidity and broader psychosocial consequences

Across the included studies, PTSD among conflict-exposed civilians in Indian-administered Kashmir was frequently reported alongside other forms of psychological distress, most commonly depressive and anxiety symptoms, in both community-based and clinical samples. Comorbidity was particularly prominent in studies involving high levels of exposure to violence or treatment-seeking populations (47, 50, 51). Direct exposure was also associated with more co-morbid symptoms (52).

Community surveys reported substantial overlap between PTSD symptoms and broader psychological distress (29, 30), while clinical samples showed high levels of psychiatric comorbidity, including depressive and anxiety disorders, among individuals presenting for care (47, 50). In one community-based study, maladaptive coping behaviours, including substance use, were reported more frequently among individuals with PTSD symptoms (50), with another reporting that direct-exposure was associated with poorer coping than indirect exposure (52).

Clinical assessments of conflict-exposed children identified high levels of PTSD symptoms, including sleep disturbance, emotional dysregulation, behavioural difficulties, nightmares, avoidance, and hyperarousal, with many cases characterised as severe at the point of clinical presentation (48). Studies focusing on orphaned children similarly reported elevated levels of psychiatric morbidity extending beyond PTSD alone (41).

A family study examining first-degree relatives of adults diagnosed with PTSD found that 62% of relatives met criteria for at least one psychiatric disorder, most commonly PTSD (32%), followed by major depressive disorder (19%) and generalised anxiety disorder (4.5%) (51).

The overall evidence suggests that PTSD in this context is closely intertwined with broader patterns of psychological distress and psychosocial impairment. The consistency of comorbidity across populations and study designs highlights the complexity of mental health outcomes associated with prolonged exposure to armed conflict.

### Chronicity and temporal course

Across the reviewed literature, PTSD associated with armed conflict in Indian-administered Kashmir was frequently described as persistent rather than transient, particularly in studies focusing on populations exposed to repeated or severe forms of violence. Both community-based and clinical studies reported patterns consistent with symptoms emerging following exposure and remaining present for extended periods among a substantial proportion of affected individuals. Notably, much research was retrospective, which limits how strongly the following findings should be interpreted.

Evidence consistent with longer-term persistence was reported in retrospective and clinical samples. In a community-based study examining adults reporting childhood exposure to conflict-related trauma, 24.4% met criteria for PTSD in adulthood, with symptoms reported as persisting beyond ten years after the initial trauma exposure (45). Clinical studies of treatment-seeking adults also documented high levels of PTSD even when reported years after trauma exposure, often accompanied by comorbid anxiety and depressive symptoms, indicating sustained clinical presentation in those seeking care (47).

Patterns indicative of early onset and sustained symptom burden were also reported in paediatric samples. In a clinical study of conflict-exposed children assessed using DSM-IV criteria, high levels of re-experiencing, avoidance, and hyperarousal were reported, with acute onset common following exposure (48). Among orphaned children assessed clinically, PTSD and broader psychiatric morbidity were also reported at elevated levels (41).

Some studies also pointed to variability in responses over time and across individuals. In adults with conflict-related injuries, a non-linear association between post-traumatic distress and post-traumatic growth, with growth outcomes most evident at moderate levels of distress and strongest within the 18 to 25-month period following trauma exposure (44). This pattern suggests that symptom severity and time since trauma may relate to adjustment processes in complex ways in conflict-affected populations. Indirect exposure also featured in the evidence base. Among healthcare professionals, secondary traumatic stress and trauma-related cognitive responses were reported in relation to occupational exposure to others’ trauma (46), indicating that psychological effects may also occur in groups exposed through caregiving roles.

The reviewed studies suggest that PTSD in this setting is often characterised by substantial symptom burden and, where measured or clinically characterised, persistence over time, particularly among populations exposed to cumulative trauma, conflict-related injury, or early-life adversity.

## Discussion

This systematic review examined the prevalence, risk factors, and correlates of PTSD among civilians in Indian-administered Kashmir. Across sixteen studies, the findings indicate that PTSD represents a substantial psychiatric burden within this population, although prevalence estimates varied considerable across studies according to population characteristics, conflict exposure, and assessment methods. Several key patterns emerged in this review: a clear dose-response relationship between trauma exposure (such as exposure to conflict, violence, injury, and displacement) and traumatic stress. Socioeconomic and demographic risk factors were also associated with PTSD, including being socioeconomically disadvantaged and experiencing conflict-related loss, particularly for women and children. Notably, PTSD also rarely occurred in isolation in this population, and was frequently co-occurring with psychiatric co-morbidities such as depression and anxiety, and broader psychosocial difficulties such as emotional and behavioural difficulties and substance misuse. The available evidence on temporal course, which was limited, also indicated that symptoms often persisted for years following trauma exposure, particularly among individuals exposed to repeated conflict-related events.

### Measurement and comparability of prevalence estimates

Variation in the reported prevalence of PTSD must be interpreted alongside differences in diagnostic criteria and assessment methods. Earlier studies largely drew on DSM-IV or DSM-IV-TR criteria, whereas more recent work included measures aligned with DSM-5. The transition to DSM-5 involved changes to the definition of qualifying trauma, the reorganisation of symptoms from three to four clusters, and an expansion from 17 to 20 symptoms, including a separate cluster addressing negative alterations in cognition and mood (2). Although there is substantial continuity between the two systems, they do not necessarily classify the same individuals as meeting criteria for PTSD. Estimates derived from DSM-IV-based and DSM-5-based assessments should therefore be compared cautiously, particularly where differences in diagnostic system occur alongside differences in population and conflict exposure.

The studies also varied in whether PTSD was assessed using a self-report screening measure or a structured diagnostic interview. The PCL-C, PCL-5 and HTQ-16 assess PTSD symptoms and may be used to identify probable cases, while instruments such as the CAPS and MINI allow diagnostic criteria to be evaluated through structured assessment. Screening-based estimates depend on the scoring method and cut-off selected and should not be treated as equivalent to clinically established diagnoses. For example, the finding that 49.8% of college students met criteria for probable PTSD was based on the study’s PCL-C cut-off score of 50, which was not reported as having been validated specifically for Kashmiri college students. Research examining the PCL has shown that prevalence estimates can vary substantially according to the threshold applied and the underlying prevalence within the assessed population (9).

A similar qualification applies to the 19% estimate derived from the HTQ-16 in the Kashmir Valley population survey. Although the instrument was translated and culturally adapted for use in Kashmir, the accompanying validation study identified too few participants with PTSD during the structured psychiatric interview to establish a Kashmir-specific HTQ-16 cut-off (57). This figure is therefore most accurately understood as an estimate of probable PTSD based on a screening threshold. Differences in diagnostic system, instrument, cut-off, reference period, and assessment format are likely to account for part of the variation between studies, although their effects cannot be separated from differences in sampling and exposure. Future research should report these methodological decisions explicitly and, where feasible, validate screening thresholds against structured diagnostic interviews within the population being studied.

### Global perspective

This review situates PTSD in Kashmir within a context of protracted and ongoing exposure rather than discrete or time-limited traumatic events. Patterns reported across studies, including dose-response relationships between cumulative trauma and PTSD, high symptom burden in both community and clinical samples, and evidence of long-term persistence, indicate distress closely tied to sustained conditions of threat.

Across the reviewed studies, greater cumulative exposure to conflict-related events was associated with higher PTSD prevalence or symptom severity, while the analysis by Housen, Lenglet, et al. (28) found that daily stressors accounted for a relatively small part of the association between traumatic exposure and symptoms of PTSD, depression and anxiety. These findings are consistent with the conceptual framing outlined earlier; direct conflict exposure remains important, while social and material conditions associated with prolonged insecurity may contribute additional psychological burden (36). Evidence from other conflict-affected civilian populations, including Afghanistan and displacement-affected settings more broadly, indicates that long-term insecurity can play an important role in sustaining mental health problems though the balance between direct trauma and ongoing stressors varies across settings (58, 59). Evidence concerning chronicity in Kashmir requires greater caution because most included studies were cross-sectional or retrospective, Nevertheless, the presence of PTSD among adults reporting earlier conflict-related trauma and PTSD within treatment-seeking samples indicates that substantial symptoms can remain present over extended periods. In a setting where threat and insecurity may continue, these findings are appropriately interpreted alongside the concept of continuous traumatic stress, which draws attention to psychological responses occurring where danger remains current or recurrent rather than being confined to a completed traumatic event (27). Individuals with limited direct exposure remain embedded in environments shaped by conditions that sustain heightened vigilance and psychological strain.

Comparable processes are documented in other conflict-exposed populations. Studies of refugees and war-affected civilians show that repeated or cumulative trauma increases vulnerability to PTSD, while research with children living under military violence demonstrates the psychological effects of sustained threat beyond formally defined traumatic events (60, 61). Similarly, exposure to or perpetrating violence in childhood is associated with notable psychosocial challenges beyond the initial period in which violence occurred (62). Evidence from post-conflict Europe further suggests that reductions in PTSD prevalence are closely linked to improvements in social stability rather than time alone (63). The persistence of distress observed in Kashmir is also consistent with models of chronic stress adaptation, including allostatic load, which describe the cumulative psychological and physiological consequences of prolonged stress activation (64). From this perspective, sustained militarisation produces vulnerability at both individual and population levels and generates a psychological burden not resolved over time.

This is reflected in the chronicity in Kashmir, which appears to be both temporal and structural where PTSD symptoms were reported years after initial exposure. Retrospective community studies documented persistence of PTSD into adulthood following childhood exposure to conflict-related trauma (45), while clinical studies described elevated levels of PTSD accompanied by comorbid anxiety and depressive disorders (47). Despite differences in study design and population, the evidence consistently points to prolonged symptom trajectories rather than spontaneous remission. Notably, much of this is based on retrospective methodology and as such, more longitudinal studies are required in order to establish the course of PTSD in this setting with more robustness.

### Patterns of vulnerability

Patterns of vulnerability are further shaped by social position. PTSD in Kashmir is distributed along gendered, developmental, and socioeconomic lines. Similar gendered patterns have been documented in other conflict-affected populations, including war-widowed women and caregivers in Afghanistan and post-conflict settings, where structural disadvantage compounds exposure-related risk (65, 66). The absence of consistent gender differences in some child and adolescent samples suggests that these disparities develop over time through social roles and structural conditions.

Socioeconomic marginalisation further compounds risk. In such settings, distress is inseparable from the conditions of daily survival. This pattern is consistent with broader accounts of structural violence, in which political and economic arrangements shape both exposure to harm and the conditions under which recovery becomes possible (67, 68). PTSD therefore appears not only as a clinical outcome, but as a manifestation of structural inequalities shaping exposure, coping capacity, and recovery.

Children and adolescents exposed to conflict within environments marked by instability and limited institutional support appear particularly vulnerable to enduring distress, especially when exposure coincides with the loss of primary caregivers. Comparable findings have been reported among conflict-affected children in South Africa and Palestine, where early bereavement and chronic insecurity shape long-term emotional and behavioural outcomes (69, 70).

Comorbidity and secondary effects extend the psychological impact of conflict beyond those directly exposed. PTSD in Kashmir frequently co-occurs with depression, anxiety, and substance use, particularly in high-exposure and treatment-seeking populations. Family studies report substantial psychiatric morbidity among relatives of individuals with PTSD, while research with healthcare professionals documents secondary traumatic stress among those providing care in conflict-affected environments (46, 51). Similar patterns have been reported in other low-resource and conflict settings, indicating that trauma circulates through relational and caregiving networks as well as through direct exposure.

### Cultural context and the assessment of trauma-related distress

The interpretation of PTSD in Kashmir requires attention to the social and cultural contexts through which distress is experienced, recognised and communicated. Most studies included in this review used DSM-derived criteria and established screening or diagnostic instruments, which capture recognised PTSD symptoms but offer less insight into how suffering is described and understood in everyday life. Qualitative research conducted across all ten districts of the Kashmir Valley found that psychological distress was commonly described as tension or *pareshani* associated with the continuing political *halaat*, or situation. Participants referred to physical, cognitive, social, and behavioural changes, including *ghabrahat*, heart palpitations, altered sleep, weakness, excessive thinking, social withdrawal, and difficulty carrying out everyday activities. Many of these experiences corresponded with items in the HTQ-16 and HSCL-25, while the authors also identified a need for a locally grounded instrument capable of capturing additional expressions of distress. Because the study examined psychological distress broadly, its findings provide contextual evidence rather than a PTSD-specific account. They nevertheless show that experiences relevant to trauma may be communicated through locally meaningful language that standard symptom categories do not fully convey (30).

Cultural and social contexts may also shape whether distress is recognised as a mental health concern, how openly symptoms are reported and where support is sought. Participants described stigma associated with mental illness and psychiatric treatment, including anticipated consequences for social acceptance, marriage and employment, alongside limited community knowledge about how to recognise and respond to psychological distress. Help-seeking commonly involved traditional or spiritual healers as well as biomedical services, captured in the expression Dua Ti Dawa Ti, or prayer and medicine. These findings concern community understandings of psychological distress and mental illness rather than PTSD specifically, and they should not be taken as evidence of a single or uniform Kashmiri response to trauma. They do, however, indicate that symptom recognition, disclosure and help-seeking occur within particular social, religious, and service contexts. Where prevalence estimates depend on self-report, these processes may affect whether experiences are recognised and communicated through the categories offered by an assessment instrument. Clinical responses may similarly be strengthened by attending to locally meaningful expressions of distress and established routes to care alongside formal diagnostic assessment (30).

These findings bear directly on the cultural validity of the measures used in the reviewed studies. A systematic review of PTSD research in India identified considerable variation in the conceptualisation and measurement of PTSD and concluded that sociocultural norms may shape its manifestation (33). In Kashmir, the HSCL-25 and HTQ-16 were translated and culturally adapted before use in the population survey. The HTQ-16 demonstrated high internal reliability, but too few participants received an interview-confirmed PTSD diagnosis for criterion validity to be established or a Kashmir-specific threshold to be calculated (57). The survey estimate of 19% should therefore be interpreted as a screening-based estimate of probable PTSD rather than as a locally validated diagnostic prevalence estimate. Future research should combine locally grounded qualitative enquiry with careful translation, assessment of semantic and conceptual equivalence, and criterion validation against structured diagnostic interviews. This would strengthen prevalence estimation and support clinical responses that recognise established PTSD symptoms alongside locally meaningful ways of understanding and communicating distress.

### Limitations

The evidence base on PTSD among civilians in Indian-administered Kashmir is affected by substantial differences in sampling and population. Firstly, this review drew on four major databases to provide broad coverage of medical, psychological, and interdisciplinary research on PTSD with no publication restrictions to maximise inclusion of relevant studies and reference lists of selected papers were also searched for additional articles. However, several limitations should be noted: the review did not include databases such as Embase or regional South Asian indices, or a systematic search of grey literature, which could potentially exclude humanitarian reports and other non-peer reviewed sources; this approach was chosen for the purposes of systematic and scientific rigour. This is particularly relevant in Kashmir, where some mental health data have been reported outside academic journals; indeed, two included studies from Médecins Sans Frontières-related projects were identified through reference screening rather than database searches (13, 14). Additionally, the review was restricted to English-language publications, meaning that the findings may not fully represent the entire body or literature on the topic.

In the included sample, several studies examined groups selected because they had experienced particularly severe exposure or were already connected to clinical or institutional settings, including treatment-seeking patients, people with conflict-related injuries, orphaned children, college students, relatives of patients with PTSD and healthcare professionals. These studies provide important evidence about populations facing pronounced vulnerability, but their estimates cannot be generalised to the wider civilian population. The larger community surveys offer a broader basis for estimating population burden, although representativeness remains difficult to establish where response rates, non-participation, and the characteristics of people outside the achieved sample are incompletely reported. The direction of selection bias may therefore differ between studies: highly exposed and treatment-seeking samples may produce elevated estimates, while incomplete coverage of marginalised or inaccessible groups may leave some experiences undocumented. Meta-analytic evidence from conflict-affected populations has similarly shown that non-random sampling, smaller samples, and self-report assessment are associated with higher estimates of PTSD and depression (71).

Recall bias creates further uncertainty where exposure was reconstructed retrospectively, particularly in studies asking adults to report traumatic experiences from childhood or events that had occurred many years earlier. Reports of traumatic exposure can vary over time, and current PTSD symptoms may influence how earlier events are recalled and reported. This does not invalidate participants’ accounts, although it limits conclusions about the timing and continuity of symptoms. For example, the identification of PTSD among adults who reported childhood trauma supports an association between recalled early exposure and later psychiatric morbidity, but a retrospective assessment cannot establish when PTSD began or whether symptoms remained continuously present across the intervening period.

The predominance of cross-sectional designs also limits insight into temporal ordering of experience, causality, and psychological processes. Associations between cumulative exposure, daily stressors, socioeconomic disadvantage, and PTSD are consistent with the possibility that repeated trauma and continuing adversity contribute to distress, although measurements collected at one time point cannot determine the direction of these relationships. Socioeconomic disadvantage may increase vulnerability to PTSD, while PTSD may also affect education, employment, and household functioning, and both may be sustained by continuing insecurity. Cross-sectional mediation findings should likewise be interpreted as statistical associations rather than evidence that the proposed mediator operated causally over time, because cross-sectional mediation models can produce biased estimates of longitudinal processes (72). The evidence therefore supports cumulative exposure and ongoing stressors as important correlates of PTSD, while prospective studies with repeated assessments are required to establish symptom onset, remission, recurrence, and change in relation to evolving political and security conditions.

Publication and selective-reporting biases may also have shaped the available evidence. Studies reporting high PTSD estimates or statistically significant associations between conflict exposure and psychological distress may be more likely to be submitted, published, and indexed than studies reporting lower estimates or null findings. The extent of this bias cannot be quantified because the studies did not provide a sufficiently comparable set of effect estimates for meta-analysis or formal assessment of funnel-plot asymmetry. Funnel-plot asymmetry would not itself establish publication bias because it may also reflect clinical and methodological heterogeneity (73). The findings should therefore be interpreted as evidence of a substantial mental health burden among the populations studied, particularly highly exposed and socially vulnerable groups, rather than as establishing a precise population prevalence, a definitive hierarchy of vulnerability factors or continuous symptom trajectories over time.

The restriction of the review to English-language publications creates a further limitation. Although the eligibility criterion was reported transparently, it may have excluded relevant studies published in other languages, including research produced or disseminated primarily within the region. The resulting synthesis may therefore provide a less complete account of the available evidence and may underrepresent findings that have not entered English-language and internationally indexed publication channels. Empirical research has not identified a consistent effect of English-language restrictions across systematic reviews, and the direction or magnitude of any resulting bias cannot be determined for the present review (74). The findings should nevertheless be interpreted as a synthesis of the English-language evidence rather than an exhaustive account of all research on PTSD among civilians in Indian-administered Kashmir. Further reviews could be strengthened by searches that include non-English publications and, where feasible, locally disseminated research.

The predominance of quantitative research also limits insight into how distress is understood, expressed, and managed within Kashmiri communities. Participatory, qualitative, and mixed-method designs could complement epidemiological research by examining lived experience, coping practices, and culturally situated expressions of distress. Furthermore, the risk of bias and quality assessment was conducted by a single reviewer. While extraction and quality assessment conducted by dual independent reviewers represent best practice, the use of a preregistered protocol, structured extraction form, and standardised quality criteria mitigated the risks typically associated with single-reviewer assessment.

Finally, it is important to note that this review focuses on Indian-administered Kashmir in accordance with its stated scope, and its findings should not be assumed to represent populations living in other parts of the wider Kashmir region.

### Implications

This review indicates that PTSD in Indian-administered Kashmir cannot be separated from its social and political context. Chronicity in this setting appears to be sustained by ongoing exposure and the absence of stable conditions that support recovery. Militarisation, surveillance, and prolonged insecurity shape daily life in ways that maintain psychological threat at a community level. As such, PTSD prevalence reflects not only individual pathology but also the operation of structural violence. Evidence from post-conflict settings such as Rwanda and Sierra Leone suggests that processes of accountability and social recognition can contribute to collective recovery by altering the conditions that sustain distress (42, 75).

From a service delivery perspective, the scale of need in Kashmir exceeds the capacity of conventional specialist-led models. With a severely limited mental health workforce relative to population size (76), community-based and task-shared approaches are likely to be necessary. Evidence from low-resource and conflict-affected settings indicates that task sharing and stepped-care models can improve access when appropriately supported (77, 78). Training community health workers and primary care staff in trauma-informed care, supplemented by specialist supervision and telepsychiatry, may help extend reach while maintaining clinical safety. Additionally, more research specifically focusing on addressing the psychiatric burden in Kashmir and other low-resource setting could help inform effective future interventions (79).

Schools represent an important site for intervention given the level of exposure among children and adolescents. School-based psychosocial programmes implemented in other conflict settings, including Palestine, suggest potential benefits for emotional and social functioning, particularly for children affected by bereavement or displacement (80). Attention to gendered dimensions of trauma is also required, alongside targeted support for healthcare workers and caregivers who are vulnerable to secondary traumatic stress in contexts of sustained insecurity (46).

Beyond Kashmir, these findings have broader relevance for global mental health. They demonstrate that low-intensity, but enduring militarisation can generate population-level PTSD comparable to acute conflicts. As forced displacement and prolonged insecurity continue to affect civilian populations globally, mental health frameworks must account for contexts characterised by ongoing threat rather than assuming a transition to post-conflict recovery (81). Addressing PTSD in Kashmir therefore requires integrated responses that extend beyond clinical care to include social protection and reduction of structural sources of harm.

## Conclusion

This review examined quantitative evidence on PTSD prevalence, risk factors, and correlates among civilians living in Indian-administered Kashmir. Across sixteen studies conducted over two decades, PTSD was reported at elevated levels in community samples, clinical populations, and specific high-exposure groups, including women, children, and those affected by cumulative or severe forms of violence. Despite variation in study design and assessment methods, the recurrence of high symptom levels across contexts suggests that PTSD represents a common feature of civilian mental health in this setting.

The findings indicate that PTSD in Kashmir cannot be understood solely as a response to isolated traumatic events. Rather, distress appears closely linked to prolonged exposure, repeated disruption, and the absence of sustained periods of safety. Symptoms were frequently persisting considerably beyond initial exposure, often accompanied by depression or anxiety, and shaped by social and economic conditions that limit opportunities for recovery. These patterns are consistent with research from other settings affected by long-term conflict, while reflecting the specific realities of civilian life under sustained militarisation. At the same time, the available evidence remains constrained by methodological limitations. Most studies relied on cross-sectional designs and screening instruments, restricting insight into symptom trajectories, change over time, and the meanings individuals attach to distress. Based on the findings in this review, future research should prioritise greater use of longitudinal and mixed-method approaches, as this would strengthen understanding of how PTSD develops, persists, and is managed within everyday life in Kashmir. Furthermore, research including different regions of Kashmir, as well as other long-term militarised settings, could offer valuable insights and comparisons points for how prevalence, risk factors, and course are shaped by the context in which they occur, particularly if also accounting for the resource- and political dimensions within such settings.

In consolidating the existing evidence, this review provides a clearer picture of PTSD as a significant and ongoing mental health concern among civilians in Indian-administered Kashmir. Addressing this burden requires attention not only to individual symptoms, but to the wider conditions that shape exposure, vulnerability, and recovery in contexts of prolonged conflict.

## Data Availability

The original contributions presented in the study are included in the article/**Supplementary Material**. Further inquiries can be directed to the corresponding author/s.
