# Supplementary material for: Post-Traumatic Stress Disorder among civilians in Indian-administered Kashmir: a systematic review
Source: Front Psychiatry. 2026 Sep 15;17:1919292. doi: 10.3389/fpsyt.2026.1919292 (PMC13619897; doi:10.3389/fpsyt.2026.1919292)
Supplement: Supplementary file 1 [file Table1.docx]

**Appendix A. Full database-specific search strategies**

Table A1. Full database-specific search strategies used in the systematic review, including search strings, filters applied, and search dates.

| **Database** | **Platform / Interface** | **Search string (verbatim)** | **Filters applied** | **Search date** |
| --- | --- | --- | --- | --- |
| APA PsycINFO | EBSCOhost | (Kashmir*) AND (PTSD OR Trauma*) | English language; no date restrictions | 28^th^ July 2025 |
| PubMed | NCBI | Kashmir* AND PTSD | English language; no date restrictions | 28^th^ July 2025 |
| ScienceDirect | Elsevier | (PTSD OR "post traumatic stress" OR "psychological distress") AND Kashmir AND ("armed conflict" OR violence OR militarization) | English language; no date restrictions | 28^th^ July 2025 |
| Scopus | Elsevier | TITLE-ABS-KEY (Kashmir AND PTSD*) | English language; no date restrictions | 28^th^ July 2025 |

***Note:*** Broader trauma-related terms were included to maximise search sensitivity; however, studies were eligible for inclusion only if post-traumatic stress disorder (PTSD) was explicitly assessed using a validated diagnostic interview or screening measure. In addition to database searching, reference lists of included studies and relevant reviews were screened to identify any further eligible studies.

**Appendix B. Methodological quality assessment of included studies (Kmet et al., 2004)**

Table B1. Summary quality scores for included studies

| **Study (author, year)** | **Study focus / population** | **Study design** | **Kmet quality score (proportion)** | **Overall quality rating** |
| --- | --- | --- | --- | --- |
| Basu & Dutta (2010) | Children exposed to terrorist attack | Cross-sectional, case–control | 0.55 | Low |
| Margoob & Ahmad (2006) | General adult population (community prevalence) | Cross-sectional household survey | 0.64 | Moderate |
| Margoob et al. (2006a) | Adult treatment-seeking PTSD | Cross-sectional, clinical | 0.45 | Low |
| Margoob et al. (2006b) | PTSD symptoms in children/adolescents (mass trauma) | Cross-sectional, clinical | 0.45 | Low |
| Margoob et al. (2006c) | Children in orphanages | Cross-sectional, clinical | 0.45 | Low |
| Margoob et al. (2008) | PTSD following amputation | Cross-sectional, clinical | 0.60 | Moderate |
| Bhat & Rangaiah (2015) | College students | Cross-sectional survey | 0.80 | High |
| Charak et al. (2014) | Adult trauma survivors | Cross-sectional, psychometric | 0.80 | High |
| Wani & Margoob (2006) | First-degree relatives of PTSD patients | Cross-sectional family study | 0.91 | High |
| Shoib et al. (2014) | Adult clinical population | Cross-sectional, clinical | 0.91 | High |
| Dar & Iqbal (2020) | Pellet-gun-injured adults | Cross-sectional | 0.82 | High |
| Yaswi & Haque (2008) | Conflict-affected adults | Cross-sectional community | 0.86 | High |
| Housen et al. (2017) | General adult population | Population-based survey | 1.00 | High |
| Housen et al. (2019) | Adult population (daily stressors) | Secondary analysis | 1.00 | High |
| Dar et al. (2015) | Adults with childhood trauma | Retrospective community-based | 0.86 | High |
| Daret al. (2024) | Healthcare professionals (secondary trauma) | Cross-sectional | 0.86 | High |
|  |  |  |  |  |

***Note***: Quality was assessed using the Standard Quality Assessment Criteria for Evaluating Primary Research Papers from a Variety of Fields (Kmet et al., 2004). Scores represent the proportion of criteria met (0-1). Items not applicable to a given study design were marked as not applicable and excluded from the total possible score for that study. Studies were classified as high quality (≥ 0.80), moderate quality (0.60–0.79), or low quality (< 0.60). Quality scores were used to inform interpretation of findings and were not used as exclusion criteria.
